# A Programmable Handheld Extrusion‐Based Bioprinting Platform for In Situ Skin Wounds Dressing: Balance Mobility and Customizability

**DOI:** 10.1002/advs.202405823

**Published:** 2024-10-22

**Authors:** Chenmin Wang, Chengwei Hu, Haojin Cheng, Weichen Qi, Liangliang Wang, Tianchi Wu, Jun Wu, Xu Cui, Jiake Xu, Haobo Pan, Shaoquan Bian, Weijia William Lu, Xiaoli Zhao

**Affiliations:** ^1^ Research Center for Human Tissue and Organs Degeneration Institute of Biomedicine and Biotechnology Shenzhen Institute of Advanced Technology Chinese Academy of Sciences Shenzhen 518055 China; ^2^ Department of Orthopaedics and Traumatology The University of Hong Kong Hong Kong 999077 China; ^3^ University of Chinese Academy of Sciences Beijing 100049 China; ^4^ Shenzhen Key Laboratory for Innovative Technology in Orthopaedic Trauma Department of Orthopaedics and Traumatology The University of Hong Kong‐Shenzhen Hospital Shenzhen 518055 China; ^5^ Faculty of Pharmaceutical Sciences Shenzhen University of Advanced Technology Shenzhen 518055 China; ^6^ The University of Western Australia Perth Western Australia 6000 Australia

**Keywords:** handheld printers, in situ bioprinting, programmable, skin regeneration

## Abstract

Bioprinting technology plays a crucial role for constructing tissue substitutes. However, the mismatched scaffold shapes and the poor treatment timeliness limit its clinical translational application. In situ printing technology that prints bioregenerants directly inside patient's body can meet the needs of specific tissue repair. This study develops a smartphone controlled handheld bioprinter for in situ skin wounds dressing. The mini bioprinter can be handheld and placed on any printing surface to create strips, complex patterns, and 3D structures, and can be equipped with microchannel needles to expand functionality. The size of the strips as well as the printing path can be programmed and controlled by the smartphone to ensure the precision of the printed product quality. Furthermore, the device not only allows for smooth switching between different bioinks for printing heterogeneous structure, but also allows for fast and uniform coverage of large wound surfaces. When dealing with complex wounds in vitro & vivo, the printer can effectively fill and precisely close wounds, promoting wound healing. The programmable handheld bioprinter can balance mobility and customizability in the management of skin wounds and is expected to realize its potential for emergency medical treatment in condition‐constrained scenarios, such as battlefields or disaster areas.

## Graphical abstract

This study presents the development of a smartphone‐controlled, programmable handheld bioprinter designed for in situ skin wound dressing. By integrating the automation of the robotic bioprinting system with the portability of the handheld device, this innovation offers a customizable scaffold that matches the wound size and provides a timely treatment option for tissue regeneration.

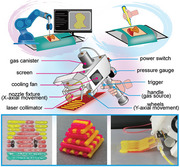

## 1 Introduction

Currently, bioprinting has become an effective tool for fabricating bioactive scaffolds, to meet the demand for tissue and organ repair.^[^ ^1^ ^]^ The process involves the composition of bioinks using suitable biomaterials, seed cells, and various growth factors, and the layer‐by‐layer deposition of these bioinks to create transplantable scaffolds based on predefined scaffold structures in software programs.^[^ ^2^ ^]^ Extensive research has demonstrated the successful generation of a range of tissue substitutes using bioprinting, including but not limited to skin, bone, cartilage, and vascular structures.^[^ ^3^ ^]^ However, the clinical translation of bioprinting still faces challenges.^[^ ^4^ ^]^ One primary reason is the lengthy post‐processing procedures required for the transition from the fabrication of scaffolds in vitro to their implantation during surgery, making it difficult to meet the immediate therapeutic needs of patients. Furthermore, there are issues with the mismatch between the printed scaffolds and the shape of the wounds. Additionally, the high investment costs and complexity of operation associated with bioprinting equipment may also hinder the clinical translation of this technology.

The emergence of in situ bioprinting aims to address these challenges. In situ bioprinting is a technology that uses bioprinting equipment to print bioinks directly at the wound based on the characteristics of the patient's injury site. It can adjust the printing scheme timely according to the dynamic changes of the wound to ensure that the implanted scaffold accurately matches the geometry of the defect. The binding of scaffolds to wound residual tissues is enhanced by in situ cross‐linking.^[^ ^5^ ^]^ Finally, in situ bioprinting uses the human body as a natural tissue culture container, which provides an ideal environment for tissue culture and reduces the chance of contamination.^[^ ^6^ ^]^ In the field of in situ bioprinting, the development of bioinks has been widely explored.^[^ ^7^ ^]^ For example, He's team has developed a bioconcrete bioink that was both portable and strong in tissue bonding, which can be used for in situ bioprinting of bone tissue.^[^ ^8^ ^]^ Injectable gel systems applied in the clinic, such as platelet‐rich plasma (PRP) systems, have also been employed as bioinks for in situ bioprinting.^[^ ^9^ ^]^ Printing equipment is also an indispensable part of in situ bioprinting, but the development of printing equipment is relatively insufficient at present.

**Figure 1:**
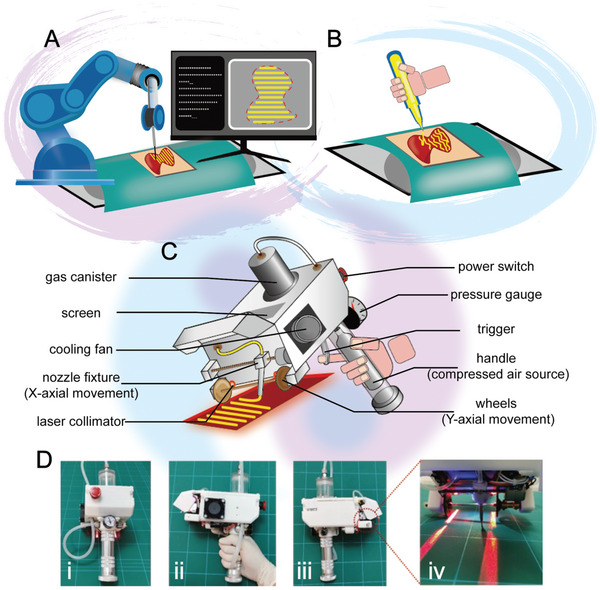
Schematic representation of the concept and structure of in situ bioprinting equipment. A) a robotic arm system under digital program guidance and B) a handheld printing device manually deposit bioinks onto tissue defects to perform in situ bioprinting. C) Illustration of the programmable handheld bioprinter dispensing bioinks in a controlled, uniform manner as directed by programmed instructions while being operated manually to fill tissue defects as needed. D) Photographs of the programmable handheld bioprinter from different orientations (The square on the table mat has a side length of 5 cm).

In situ bioprinting equipment can be categorized into digitally programmed robotic arm systems (**Figure** **1**A) and handheld devices (Figure 1B). The robotic arm system utilizes 3D scanning technology and algorithms to create real‐time optimized printing trajectories, allowing precise distribution of bioinks to the wound.^[^ ^10^ ^]^ For example, De Maria's team has designed a robotic bioprinting system equipped with path planning algorithms. This innovation enabled the printing nozzle to navigate closely through the irregular contours of bone defect models, effectively filling the voids with bioinks.^[^ ^11^ ^]^ As for handheld bioprinting devices, clinicians can manually apply bioinks to the patient's wound. For instance, Zhang's team has introduced a low‐cost, open‐source handheld extrusion printer integrated with UV illumination, which can distribute bioinks to skin wound and initiate cross‐linking for immediate solidification.^[^ ^12^ ^]^ The robotic arm system reduces manual intervention and achieves higher automation and printing precision through program guidance. However, these devices currently face challenges such as their relatively large size and the complexity of specialized operation, limiting their widespread clinical application. Handheld printing devices avoid the complex steps of 3D scanning and digital reconstruction, lowering the technical threshold, and offering compact portability and flexible operation. Nevertheless, the printing accuracy and stability are relatively lacking.^[^ ^13^ ^]^

In order to provide a printing device that balances both printing accuracy and convenience in in situ bioprinting, we developed a smartphone‐controlled programmable handheld printer in this study. This portable printer is easy to use, the user can hand‐control it to reach the target area, the nozzle performed custom printing task under programmed instructions (Figure 1C,D). The printer can adapt to different viscosity ranges of bioinks, and by adjusting the printing parameters to effectively control the printed strip structure characteristics. On this basis, with the help of the printing programming module, a variety of patterns can be personalized design and printing. In addition, when combined with microchannel nozzles, the printer also has an ability to print strips of multiple bioink combinations, enabling gradient structure and efficient coverage over large areas. It is worth emphasizing that we also explored the use of the printer for in situ bioprinting on skin wounds with a variety of complex conditions to evaluate its potential for accurate and rapid transplantation as well as promoting wound healing.

## 2 Results and Discussion

### 2.1 Design and Manufacture of the Programmable Handheld Printer

This study designed and manufactured a programmable handheld printer (Figure 1C,D). This portable printing device can be handheld transferred to the target area and fixed. Guided by a preset program inside a smartphone, the nozzle prints the bioinks precisely on the specific area to form a predetermined strip pattern. The operation of the printer depends on three main modules: a control module, a bioinks extrusion module and a printing path module. The control module consists of a smartphone that can connect to the printer via Wi‐Fi, and its Web App can set multiple printing parameters such as printing path and speed and generate corresponding program instructions. The bioinks extrusion module incorporates an internal pneumatic system and an external mechanical system, where the driving pressure of the pneumatic system exhibits a highly linear relationship with the program instructions (Figure S1, Supporting Information). As for the printing path module, it moves precisely in the printing plane according to the program instructions through the nozzle fixed to the fixture and the orthogonal mechanical transmission device. At the same time, the program setting of the printing speed is also accurately corrected with the actual printing speed (Figure S2, Supporting Information).

**Figure 2:**
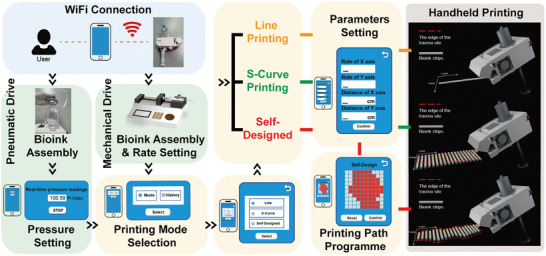
Illustration of the operational workflow of the programmable handheld printer. From left to right, the process initiates with a smartphone connecting to and controlling the printer via Wi‐Fi. This is followed by the selection of either a pneumatic or mechanical extrusion method; setting the corresponding printing parameters; and loading the bioink. Subsequently, the user selects the desired mode in the Web App's printing path planning interface, which generates the program instructions for printing. Finally, the printer is manually operated to execute the printing task.

In situ bioprinting, the operation process is shown in **Figure** **2**. The printer first connects to the smartphone control via Wi‐Fi. Then, according to the characteristics of bioinks and the requirements of printing strip, the appropriate extrusion method is selected. In the pneumatic extrusion mode, the smartphone Web App interface can monitor the pressure applied to the bioinks in real time. For mechanical extrusion, the extrusion rate and duration can be customized through the micro‐extrusion pump. Next, the user can select three preset printing paths in the Web App (Movie S1, Supporting Information). In the Line Printing mode, the operator uses a trigger to control the continuous extrusion of bioinks to form the desired line strip. In the S‐Curve Printing mode, the printer first lays down a transverse strip, then advances a set distance longitudinally as driven by the rollers, and proceeds to print the next transverse strip, continuing this pattern to ultimately achieve S‐shaped strips of bioink on the plane. In the Self‐Design mode, users can customize the design pattern in the interface of the Web App. The generated program instructions control the printer to distribute the bioinks automatically and precisely in the selected area, creating a personalized pattern.

This printer is designed for compatibility and ease of operation. The choice of two bioink extrusion methods and the adjustability of printing parameters ensure that it can adapt to different characteristics of bioinks. The Web App interface design on the smartphones is intuitive and simple, which greatly simplifies the operation process and reduces the threshold of user use.

### 2.2 Printing Performance Evaluation of the Programmable Handheld Printer

**Figure 3:**
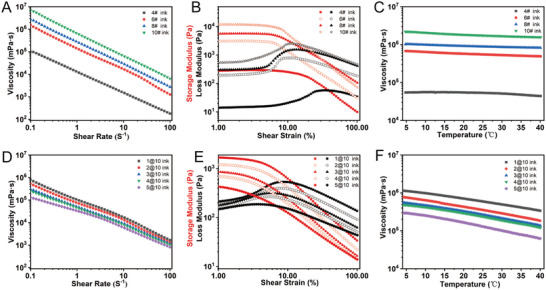
Rheological evaluation of two bioink systems. A–C) depict the shear‐thinning behavior of the laponite XLG‐based bioink system under shear rates ranging from 0.1 to 100 s^−1^, the oscillatory shear test across shear strains from 1% to 100%, and the temperature sensitivity evaluation within the range of 4 to 40 °C, respectively. D–F) correspond to the granular hydrogel bioink system, presenting the same tests for shear‐thinning behavior, oscillatory shear tests, and temperature sensitivity evaluation under identical testing conditions.

To fully evaluate the printing performance of the newly developed programmable handheld printer, two bioink systems with rheological differences were prepared for testing. As we all know, in the field of bioprinting, a wide variety of bioink systems play a crucial role, and the characteristics of the bioink must be matched to the needs of the tissue to be repaired.^[^ ^14^ ^]^ Taking bone tissue damage repair as an example, scaffolds with high mechanical strength are usually chosen in bioprinting applications because they are more conducive to the reconstruction of bone.^[^ ^15^ ^]^ Among them, high‐strength scaffolds made from bioinks with nano‐clay additives such as laponite XLG are a common choice for repairing bone defects.^[^ ^16^ ^]^ On the other hand, for tissues such as skin, nerves and musculoskeletal, soft tissue scaffolds prepared by using low‐viscosity bioinks are more conducive to the normal differentiation of cells and their physiological functions.^[^ ^17^ ^]^ Scaffolds based on a bioink system of granular hydrogels, for example, are typically both soft and flexible.^[^ ^18^ ^]^ Therefore, in this study, we chose viscosity as the main evaluation criterion and prepared two kinds of bioinks (**Figure** **3**): one was a bioink system based on Laponite XLG with a relative high viscosity in the range of 9.95 × 10^4^ and 6.85 × 10^6^ mPa s (shear force of 0.1 s^−1^); The other was a lower viscosity granular hydrogel bioink system based on hyaluronic acid (HA) and polyethylene glycol diacrylate (PEGDA), with a viscosity between 1.33 × 10^5^ and 8.00 × 10^5^ mPa s (shear force of 0.1 s^−1^). Both bioinks can undergo a gel‐sol transition under shear forces. With these two bioinks, we aimed to evaluate the adaptability and printing effectiveness of the printer for bioinks with different viscosity ranges.

**Figure 4:**
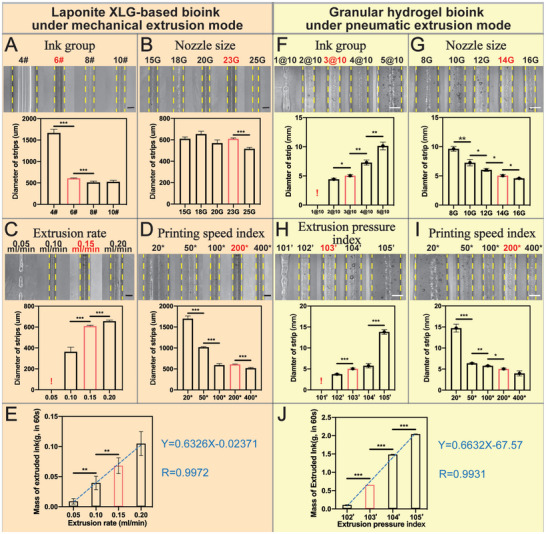
The performance of the strips printing by the programmable handheld bioprinter. A–D) Strip width variations with Laponite XLG‐based bioink compositions, nozzle sizes, extrusion rates and printing speed index under mechanical extrusion mode; scale bar = 500 µm. E) Mass of Laponite XLG‐based bioinks extruded per 60 s at various extrusion rates under mechanical extrusion mode, and its linear fitting was performed. F–I) Strip width variations with granular hydrogel bioink compositions, nozzle sizes, extrusion pressure index, and printing speed index under pneumatic extrusion mode; scale bar = 5 mm. J) Mass of granular hydrogel bioinks extruded per 60 s at various extrusion pressures index under pneumatic mode, and its linear fitting was performed. (Notes: The shared control group is highlighted in red. !: Discontinuous strips were excluded from the statistical analysis. *p* < 0.05 (*), *p* < 0.01 (**), and *p* < 0.001 (***)).

The programmable handheld printer adopted a mechanical extrusion mode to print the S‐Curve path of the Laponite XLG‐based bioinks (**Figure** **4**A–D). The results showed that increasing the nano‐clay content in the Laponite XLG‐based bioink and increasing the printing speed could reduce the strip width, while decreasing the nozzle diameter had a relatively limited effect on the strip width. At lower extrusion rates, the bioinks based on Laponite XLG were difficult to form continuous and stable strips, but the printed strips became significantly thicker as the extrusion rate increased. Granular hydrogel bioinks were used to evaluate the pneumatic extrusion mode of the printer (Figure 4F–I). When the liquid phase component ratio of the granular hydrogel bioinks or the extrusion pressure was low, the gas pressure was not enough to achieve continuous and uniform extrusion of the bioinks. However, with the increase of liquid component ratio and extrusion pressure, the strip width also increased significantly. At the same time, the influence of nozzle diameter and printing speed on strip width was also obvious: with the decrease of nozzle diameter and the increase of printing speed, the width of strips was significantly reduced. In the mechanical extrusion mode, the strip width was adjusted in the range of ≈0.40 to 1.70 mm, while the pneumatic extrusion mode can significantly widen the strip width in the range of ≈4.0 to 15.0 mm. It is worth noting that, in terms of extrusion stability, both pneumatic and mechanical extrusion modes showed a highly linear correlation between the amount of bioinks extruded and the drive power (Figure 4E,J).

In summary, it was confirmed that the printer can in situ print for bioinks with different viscosity ranges under pneumatic and mechanical extrusion modes and can obtain strips with specific characteristics according to different printing parameters. The printer's mechanical extrusion mode can meet the precision requirements of in situ bioprinting, while the pneumatic extrusion can efficiently fill and cover a large area of damage, demonstrating its potential to match the needs of different therapeutic scenarios. In addition, the results of this study also had important guiding value for the subsequent print path planning and parameter setting of personalized pattern distribution.

### 2.3 Programmed Instruction‐Controlled Handheld Printing for Bioinks Distribution of Voxelization

**Figure 5:**
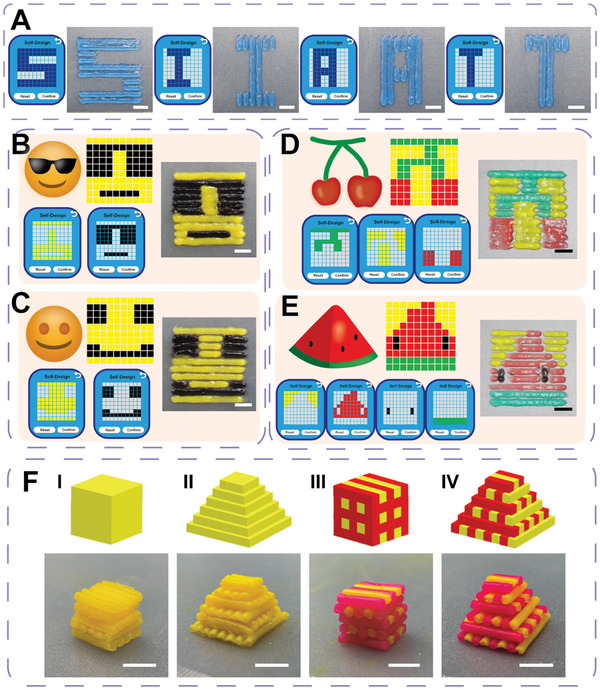
Demonstration of programmable handheld printing for mono/multi‐bioink distribution of voxelization. A) Pixelated “S”, “I”, “A”, “T” characters were input into the Web App's Self‐Designed path planning interface to generate corresponding program instructions, directing the programmable handheld printer to print granular hydrogel bioinks and form a monochrome pattern. B–E) Building upon the mono‐bioink pattern, color images were broken down into multiple printing channels by color and programmed individually. Sequential programmable handheld printing of each channel's granular hydrogel bioink formed complex smiley face and fruit patterns composed of multiple bioinks. F) Using Laponite XLG‐based bioink and manual adjustment of the programmable handheld printer's position facilitated the printing of mono‐bioink cubes (I) and pyramids (II), as well as bi‐bioink cubes (III), and pyramids (IV).

The tissues and organs exhibit a high degree of complexity, which is mainly reflected in the structure diversity and the component spatially heterogeneous distribution.^[^ ^19^ ^]^ Tissues such as skin and cartilage, for example, are histologically stratified, and there are significant differences in composition between different layers.^[^ ^20^ ^]^ On this basis, it is particularly important to form regenerative fillers in the damaged site by in situ bioprinting that are consistent with the biological characteristics of the damaged tissue. This requires precise regulation of the spatial distribution of bioinks and a variety of bioinks with different compositions and functions to participate in the printing process. The evaluations of the ability of the programmable handheld printer to create personalized manufacturing of complex patterns and structures, using the bioink systems mentioned above, have be conducted. For example, in the Self‐Designed option, input the character pattern that needed to be printed – “S”, “I”, “A”, “T”. The program generated by the Web App would guide the printer nozzle to extrude bioinks in the specified area of the printing platform, and finally form the exact pattern of “SIAT” (as shown in **Figure** **5**A; and Movie S1, Supporting Information). The proper positioning accuracy of the printer made it possible to create colorful patterns using multiple bioinks. These multicolor patterns can be broken down into multiple print channels depended on color, each of which can be independently programmed to generate corresponding printing instructions. This process resulted in the precise presentation of complex patterns, such as bicolor smiley faces (Figure 5B,C) and multicolor fruit patterns (Figure 5D,E). In addition, thanks to the flexibility of the handheld printer, after printing the bottom layer, the operator can manually adjust the nozzle to the appropriate height to overlay additional layers of structure on top of the existing pattern. From this, we can customize cube and pyramid shapes in monochrome and multicolor (Figure 5F).

These patterns, generated by the programmable handheld printer, showed an orderly and regular arrangement of strips, that closely correspond to the preset layout scheme on the smartphone interface. In the multicolor printing process, the bioinks of each color channel were precisely embedded in the corresponding position, avoiding obvious overlap and distortion, ensuring the clarity of the pattern and the accuracy of the color. In general, the product quality of handheld printing equipment is highly dependent on the technical proficiency and experience of the operator, which can lead to unstable printing quality and difficult to ensure consistency.^[^ ^21^ ^]^ In contrast, the design concept of programmable handheld printing is to balance the mobility and customizability, thereby minimizing the impact of human error on the printing results. With the addition of programmable control functions, the printer showed satisfactory repeatability in positioning accuracy, ensuring the stability and reliability of the printing process, and providing robust control for in situ bioprinting.

### 2.4 Programmable Handheld Printing Bioinks Combinations through Microchannel Nozzles

**Figure 6:**
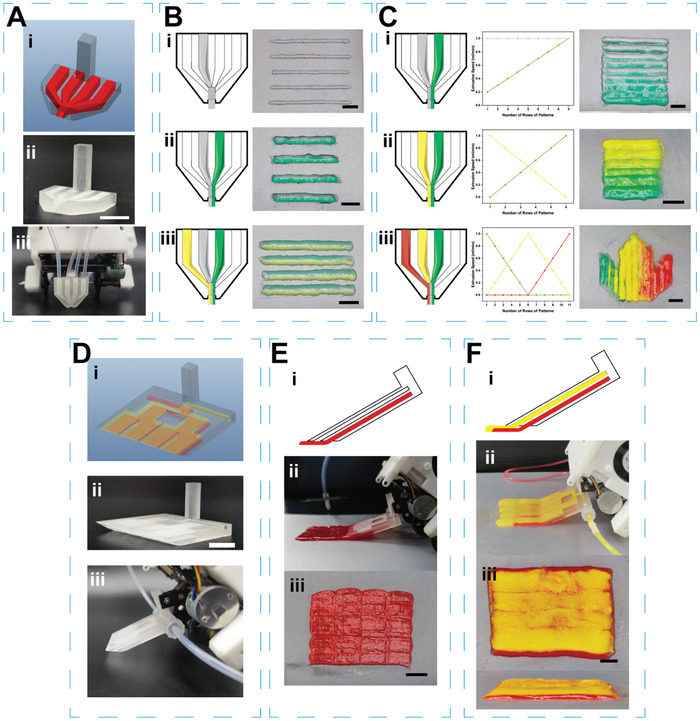
Printing with microchannel nozzles on the programmable handheld printer. A) Displayed from top to bottom the 3D rendering, actual image, and the nozzle attached to the printer for the “all‐in‐one” microchannel nozzle. B,C) Illustrated the bioinks distribution within the “all‐in‐one” microchannel nozzle during the printing of mono and multi‐bioink, with corresponding photographs of the resulting strips. The transparent color represented granular hydrogel bioinks extruded pneumatically, while other colors indicated laponite XLG‐based bioinks extruded mechanically. The dot plots in (C) indicated the extrusion rates of different colored bioinks during printing of various rows of patterns. Scale bar = 1.00 cm. D) Displayed from top to bottom the 3D rendering, actual image, and the nozzle attached to the printer for the “thin‐layer” microchannel nozzle. E,F) Showed the bioinks distribution diagrams within the “thin‐layer” microchannel nozzle during mechanical extrusion of single and double layers of laponite XLG‐based bioinks, along with photographs of the corresponding thin layers produced. Scale bar = 1.00 cm.

As mentioned above, the printing of individual bioinks one by one is one of the ways to achieve multi‐bioink patterns and is widely used in layer‐by‐layer printing of tissue substitutes with characteristics such as gradient structure.^[^ ^22^ ^]^ This is a frequently encountered problem in bioprinting, and the typical solution is to increase the corresponding bioink cartridges and nozzles, and to switch the nozzles repeatedly during the printing process. This affects the printing efficiency of multiple bioinks on the one hand, and on the other hand raises the complexity of the device, including the requirement for repeatable positioning accuracy during nozzle switching. In order to improve efficiency, nozzles with microchannel features have been gradually introduced to achieve efficient printing of multi‐bioink structures.^[^ ^23^ ^]^ With the use of microchannel nozzles, bioinks from different channels can be freely combined and extruded under precise program control, enabling instant switching and diversified output of bioinks, greatly expanding the functionality of the printer. For example, the “all‐in‐one” microchannel nozzle equipped on the programmable handheld printer in **Figure** **6**A has four interconnected channels, allowing the simultaneous introduction of multiple bioinks to form a multi‐colored composite stripe at the outlet, as shown in Figure 6B. By adjusting the extrusion ratios of bioinks in each channel, continuous variations in the proportions of bioinks during the printing process can be achieved without the need to change the nozzle, creating printing patterns with unidirectional gradients and multi‐gradient structures, as shown in Figure 6C. This “all‐in‐one” nozzle aims to enhance handheld printing for in situ fabrication of scaffolds with different component and structural gradients. Each channel's bioink can serve as a carrier for cells or drugs and can be customized and incorporated into the bioink system of the scaffold according to specific requirements, facilitating the creation of an environment favorable for tissue regeneration. Figure 6D–F demonstrated another type of “thin‐layer” microchannel nozzle equipped on the printer, which can uniformly extrude bioinks in a thin‐layer form. By precisely controlling the extrusion rate of bioinks and the Y‐axis movement speed of the printer, efficient single‐layer or double‐layer coverage of bioinks can be achieved over a wide range. Compared to the line‐by‐line extrusion printing of in situ bioprinting, printers equipped with “thin‐layer” microchannel nozzles demonstrated efficiency in producing large‐area and even multi‐layer coatings, effectively alleviating the low efficiency issue in the fabrication of tissue scaffolds for clinical translation.^[^ ^24^ ^]^ This progress showed great potential in practical applications such as the treatment of large‐area skin wounds.

### 2.5 Programmable Handheld In Situ Bioprinting for Skin Wound Treatment

**Figure 7:**
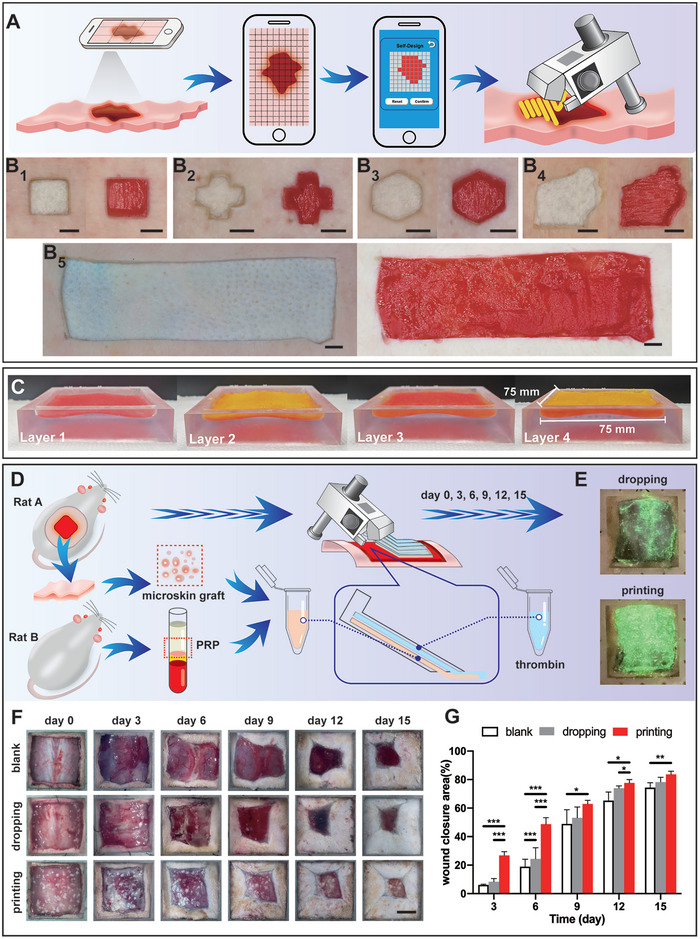
Programmable handheld in situ bioprinting for skin wound treatment. A) Schematic of the process for programmable handheld in situ bioprinting on in vitro porcine skin, including steps for wound image capture, gridding of the image, generation of printing program instructions, and bioprinting the granular hydrogel bioink to cover the wound B) Before and after images of programmable handheld in situ bioprinting on in vitro porcine skin wounds in various shapes: square (B_1_), cross (B_2_), hexagon (B_3_), random (B_4_), and a large‐area rectangle (B_5_). Scale bar = 1.00 cm. C). The programmable handheld bioprinter with a “thin‐layer” microchannel nozzle was used to print four layers of 3@10 bioink onto the simulated human full‐thickness skin wound model, effectively closing the “wound”. Different colored pigments were added between adjacent layers of bioink to distinguish each layer visually. D) Illustration of the in situ bioprinting process for a rat full‐thickness skin wound model using the “thin‐layer” microchannel nozzle, including preparing bioinks (PRP mixed with microskin grafts), in situ bioprinting of the bioinks, and post‐surgical care and observation of the rats. E) Comparison of bioinks distribution on rat skin wounds using droplet method versus programmable handheld printing, with green fluorescent dye added to the bioinks for visibility. F) Photographs depicting the healing status of rat skin wounds treated with different methods; scale bar = 1.00 cm, with wound closure rates quantified in G). (*p* < 0.05 (*), *p* < 0.01 (**), and *p* < 0.001 (***)).

Based on the above studies, the printer was further used to evaluate its potential for in situ bioprinting, specifically for the treatment of skin lesions to achieve tissue regeneration. Successful implementation of in situ bioprinting first requires rapid and accurate determination of the spatial extent of tissue defects, followed by the planning of printing paths according to these ranges and the utilization of printers for rapid filling and precise sealing treatment. Proof of concept in vitro experiments on porcine skin demonstrated that this programmable handheld printer could adapt to a variety of irregular skin wound shapes to achieve effective filling of in situ grafts (**Figure** **7**A). The specific operation was to take a photo of the wound on the porcine skin through a smartphone and grid the wound image at the appropriate proportion. In the Self‐Designed interface of the Web App, the printing path was marked according to the position of the grid covering the wound. Then, after the program instruction was generated, the handheld printer applied bioprinting layers of bioinks to the wound according to the program to close and repair the wound (Figure 7B). The hydrogel layer formed by the bioink adhered firmly to the surface of the fresh moist porcine skin wounds and did not detach during skin stretching (Figure S4, Supporting Information). Additionally, this printing method was also applicable for in situ bioprinting with cell‐laden granular hydrogel bioink (Figure S6, Supporting Information). Cells were extruded out with the bioink and deposited in the printed constructs, maintaining a high viability (79.1 ± 3.2%), thereby providing a pathway for cell therapy in damaged tissues. Microbial contamination was not found during the printing process.

Moreover, the repair of full‐thickness skin injuries in humans requires addressing deeper wounds, which can reach up to 5 mm.^[^ ^26^ ^]^ In a model simulating the irregular shape of human full‐thickness skin wounds(Figure S5, Supporting Information), the handheld bioprinter equipped with the “thin‐layer” microchannel nozzle was used to rapidly and uniformly print multiple layers of bioink (Figure 7C). This demonstrated the effectiveness of the handheld bioprinter in managing complex wounds and providing high‐quality wound filling treatment.

In practice, irregularities in the injury site may cause the flow and slippage of low‐viscosity bioinks during application, resulting in uneven coverage or bioinks leakage outside the injury site, leading to wastage (Figure 7E). To address this challenge, this study evaluated the printing capability of the printer with microchannel nozzles on a full‐thickness rat skin wound model, particularly in the raised area on the back (Figure 7F). In wound treatment, it is essential to consider the requirements for breathability and water permeability of the wound dressings. By selecting appropriate bioinks and composites, different types of wounds and their various stages of repair can meet specific needs for air and moisture exchange.^[^ ^25^ ^]^ Considering that PRP mixed with autologous microskin grafts has been widely used in clinical skin wound treatment,^[^ ^9^ ^]^ we selected this combination as the printing bioink. Using the printer equipped with a “thin‐layer” microchannel nozzle, we uniformly applied the bioink to a 2.5 cm × 2.5 cm square wound on the rat's back. The lower channel of the nozzle injected a substance mixed with microskin grafts and PRP, while the upper channel injected a thrombin solution. The substances flowing out from the two independent channels mixed stably at the outlet and rapidly formed a gel, achieving efficient and uniform coverage. In comparison, applying microskin grafts/PRP bioinks on the wound using the dropwise method often failed to achieve uniform distribution, causing the microskin grafts in PRP to tend to accumulate in the low concave areas of the body and have less distribution in the central raised portion. When using the same amount of bioink, the area covered by the dropwise method was relatively limited, weakening the potential of seed cells to repair the entire wound area. This difference explained why the healing rate in the dropwise group was generally lower than in the printing group (Figure 7G), especially in the early healing stage, with a closure rate of 26.89 ± 2.54% in the printing group on the third day, compared to only 8.37 ± 2.26% in the dropwise group, slightly higher than the 6.12 ± 0.48% in the blank control group. Additionally, based on the wound images (Figure 7F) and histological evaluation (Figure S7, Supporting Information), the wounds of rats treated with the handheld in situ bioprinter showed no signs of pathogen infection, such as redness, pus formation, or delayed healing. The H&E staining of the wound tissue also revealed no evidence of infectious inflammation. Preventing microbial infection is important in the treatment of skin wounds. Beside the clinical standardized aseptic procedures, it is suggested that adding antibacterial and antiviral agents into the bioink for better anti‐infection effect.

Overall, this programmable handheld printer combines user‐friendliness with adaptability for complex tissue printing. The printer is compact, easy to operate, and does not rely on complex equipment and techniques such as professional 3D scanning, imaging reconstruction, and slicing. The operators only need a simple training to work with the printer through the Web App on their smartphones. In comparison to the manual deposition approach, the programmable handheld bioprinter demonstrated a more uniform and precise coating of the wound surface, highlighting its ability to achieve direct, efficient, and accurate transplantation of tissue repair materials at the wound site during in situ bioprinting. Therefore, this technology streamlines the process from the laboratory to the operating room, making it particularly suitable for resource‐limited battlefield environments and remote areas, broadening new possibilities for the emergency treatment and regeneration of tissue injuries.

In summary, this study presented a novel in situ printing method that integrated a combination of handheld operation and programmed control. This unique programmable handheld printer was able to tailor the printing path to the specific morphology of irregular wounds, maintaining the flexibility of handheld printing while automating programmed printing. In addition, together with the microchannel nozzles, this device not only allowed the free switching of multiple bioinks to accommodate heterogeneous tissues during the printing process, but also provided fast and uniform multi‐layer coverage of large wounds, which was demonstrated in animal experiments on in vitro and vivo skin wound treatment. However, considering the miniaturization of the device and the requirement for cost control, this printing device has streamlined some of its functional modules, such as printing temperature control, Z‐axis drive and vibration damping module. This makes it fall short in terms of resolution and stability of printed strips compared to conventional bioprinters. Future work will focus on optimizing the design of the printer architecture to add ancillary functions and improve printing accuracy, further advancing the prospects for clinical applications of this technology.

## 3 Conclusion

This study introduced a novel concept of in situ bioprinting, merging handheld printing with programmable control processes. We have developed a programmable handheld printer operable via smartphone, accommodating a broad spectrum of bioink viscosities through both mechanical and pneumatic extrusion modes. Through the manipulation of printing parameters, the characteristics of the printed strips can be flexibly modulated. The programmable capabilities facilitated the automated and consistent creation of precise mono‐bioink, multi‐bioink, and multilayered structural patterns during handheld operation. When outfitted with microchannel nozzles, this printer can achieve advanced functions such as gradient and high‐speed printing across large areas, making it versatile for diverse applications. In the context of skin wound treatments, both in vitro and in vivo, the printer has demonstrated efficacy in sealing and uniformly covering wounds, underscoring its unique value as an innovative medical device in tissue regeneration. In essence, this printer integrated the portability of handheld devices with the precision of automated programmable printing. Its modular design allowed for the selective replacement of bioink drive modules, fluidic systems, and control devices, rendering it ideal for emergency scenarios in resource‐scarce settings, such as battlefields and remote locations. Furthermore, the integration of smartphone applications for controlling the printer streamlined the traditionally complex tasks of CAD modeling, path planning, and parameter configuration in bioprinting, thereby diminishing the learning curve and enhancing its accessibility to medical professionals. We believed that this programmable handheld printer harbored immense promise for applications in tissue regeneration medicine, biological research, and education, among other fields.

## 4 Experimental Section

### Hardware Assembly of the Programmable Handheld Bioprinter

The hardware of the programmable handheld printer was composed of three primary modules: 1) Bioinks Extrusion Drive Module. The printer incorporated a pneumatic extrusion drive system internally, consisting of a food‐grade compressed carbon dioxide gas source (≈6 MPa) located within the handle. This was connected in sequence to a medical‐grade silicone tube, an electromagnetic valve (DC6V), a 110 mL air canister, and an ink cartridge. Alternatively, a modified micro‐extrusion pump (XMSP‐2C) could be directly connected to the ink cartridge, employing a mechanical drive to extrude bioinks. 2) Printing Path Drive Module. On the X‐axis, a compact stepper motor propelled the nozzle fixture to move at a constant speed in either the positive or negative direction, with a maximum travel range of 50.00 mm. In the Y‐axis direction, another stepper motor drived a pair of parallel wheels with a diameter of 28 mm to roll at a set speed along the Y‐axis. 3) Control Module. On one hand, an upstream smartphone established a Wi‐Fi link with the printer's microcontroller (ESP32‐S2) system's Internet Protocol (IP) address 192.168.4.1, receiving program instructions from the smartphone's Web App. On the other hand, the microcontroller system controlled the activation and pausing of the bioinks extrusion drive module and executed the movements in the X and Y‐axis directions within the printing path motion module.

### Printing Speed Calibration

The printing nozzle was mounted onto the printer fixture, and after initiating the printer's operating program, speed indices of 20*, 50*, 100*, 200*, and 400* were input into the Web App's printing speed parameter option. The distance covered by the printing nozzle and the corresponding time for each speed index were recorded. The calibration test for each printing speed index was repeated five times. The collected data were analyzed to establish a correlation between the printing speed indices and the actual printing speed.

### Gas Pressure Calibration

Following the initiation of the printer's operating program, various printing pressure indices ranging from 101′ to 110′ were entered at random into the pressure parameter option. A digital pressure gauge (DLX‐DMG512B) was used to measure the corresponding output pressures, which were recorded for analysis. These data would be utilized to correct the relationship between pressure indices and actual gas pressures.

### Bioinks Preparation

Preparation of the granular hydrogel bioinks involved the following steps. For component A, 0.05 g of HA (Freda, Mw: 800–1300 kDa) and 0.5 mL of PEGDA (Sigma, Mn: 575 Da) were dissolved in deionized water containing 0.5 wt.% lithium phenyl‐2,4,6‐trimethylbenzoylphosphinate (LAP, Aladdin) to prepare a 10 mL solution, referred to as 10 parts by volume of component A. For component B, 1 mL of PEGDA were dissolved in deionized water containing 0.5 wt.% LAP to form a 10 mL solution. This solution was then exposed to a 60 W ultraviolet lamp for 10 s to initiate the polymerization reaction and form a hydrogel. The hydrogel was extruded and crushed repeatedly in a 30 mL syringe to create particles, a process that was repeated 10 times, yielding particles referred to as 10 parts by volume of component B. To prepare the granular hydrogel bioinks, various volumetric ratios of component A (1, 2, 3, 4, or 5 parts) to component B (10 parts) were mixed, resulting in bioinks labeled as 1@10, 2@10, 3@10, 4@10, and 5@10, respectively. For the preparation of laponite XLG‐based bioinks, 0.4, 0.6, 0.8, and 1.0 g of clay (Laponite XLG, BYK) were each added to 10 mL of deionized water. The mixtures were vigorously stirred for ≈1 min to ensure full hydration and the absence of significant solids, thus forming laponite XLG‐based bioinks, designated as 2#, 4#, 6#, and 10# inks, respectively.

### Bioinks Rheology Characterization

A modular compact rheometer (Anton Pear MCR 302) was employed to evaluate the rheological characteristics of both low‐viscosity and high‐viscosity bio‐inks used in this study. A plate with a diameter of 50 mm and a gap distance of 0.5 mm between parallel plates was utilized. The shear thinning behavior of the bioinks was characterized using the viscosity curve measurement mode of the rheometer, conducted at room temperature (25 °C). Shear rates were scanned within the range of 0.1 to 100 s^−1^, and the corresponding viscosities of the samples were recorded. The viscoelastic properties of the bioinks were characterized using the amplitude sweep mode of the rheometer, performed at room temperature (25 °C). A frequency of 1 Hz was applied, and shear strains were scanned within the range of 0.1 to 100%, with the resulting storage modulus (G') and loss modulus (G″) of the samples recorded. The temperature sensitivity of the bioinks was assessed using the temperature sweep mode of the rheometer. A shear rate of 0.1 s^−1^ was applied, and temperatures were scanned within the range of 4 to 40 °C at a heating rate of 5 s °C^−1^, with the corresponding viscosities of the samples recorded.

### Printing Performance Testing

A strip assay was conducted to evaluate programmable handheld printing using mechanical drive extrusion mode for the laponite XLG‐based bioinks and pneumatic drive extrusion for the granular hydrogel bioinks. For the mechanical drive extrusion of the laponite XLG‐based bioinks, four printing parameters were selected to assess their impact on the width of the printed strips, specifically the ratio of bioinks composition, nozzle size, extrusion rate, and printing speed index. (The internal diameters of 15, 18, 20, 23, and 25 G nozzles are 1.43, 0.92, 0.61, 0.34, and 0.26 mm, respectively). There was a set of reference parameters consisting of 6# ink, a 23 G nozzle, an extrusion rate of 0.15 mL min^−1^, and a printing speed index of 200* was selected. In the same way, for the pneumatic drive extrusion of the granular hydrogel bioinks, four printing parameters were selected to assess their impact on the width of the printed strips, specifically the ratio of ink composition, nozzle size, extrusion pressure, and printing speed index. (The inner diameters of 8, 10, 12, 14, and 16 G nozzles are 3.50, 3.00, 2.30, 1.60, and 1.20 mm, respectively). The parameter combination of 3@10 ink, a 14 G nozzle, an extrusion pressure index of 103′, and a printing speed index of 200* were selected as the reference group. All strips were printed and subsequently photographed under a stereoscopic microscope. In both the mechanical and pneumatic drive extrusion modes, the printer was operated continuously, and the mass of the printed strips was measured every 60 s. The extrusion rate or extrusion pressure parameter was varied, and the corresponding strip mass values were recorded. All data mentioned above were collected in triplicate for statistical analysis. All bioinks were printed at room temperature, and the temperature of the bioinks remained stable with no significant changes during the printing process.

### Microchannel Nozzles Design and Fabrication

The 3D structures of microchannel nozzles (“all‐in‐one” and “thin‐layer” nozzles) were created in part mode using Pro/ENGINEER Wildfire 5.0. Subsequently, the models were exported in STL file format. The parameters for controlling deviations were set with a chord height and angle control of 0.01. The microchannel nozzles were printed using a commercial stereolithography 3D printer (Soonser Smart‐400 Pro). The printing ink, Soonser S‐GR4002 biocompatible photopolymer, was selected. The layer thickness for printing was set at 0.1 mm, and the duty ratio of the laser (355 nm) was set to 95%.

### Porcine Skin Wound Repair In Vitro

Fresh porcine skin from the market was cleaned to remove blood clots and other impurities from the surface. After cleaning, residual moisture on the surface was wiped away using absorbent paper. A laser engraving machine (Wainlux‐JL4) was used to perform specific shape ablation on the surface of the porcine skin with a laser power of 10 W. Once the ablation process was completed, the surface char layer was carefully cleaned to expose the subcutaneous tissue, creating a skin wound with a specific shape. Subsequently, a smartphone was used to photograph the wound from directly above, with a grid overlay added to the image. The parallel grid lines were spaced 0.50 cm apart. Based on the grid coverage area over the wound, the corresponding squares were marked within the Self‐Designed printing path planning interface of the Web App, which then generated printing program instructions. Under the guidance of these printing program instructions, the granular hydrogel bioink was printed onto the wound using the programmable handheld printer's pneumatic extrusion mode.

### Cell Bioprinting

Under sterile environment, the 3@10 bioink was prepared, using PBS as the solvent. Human umbilical vein endothelial cells (HUVECs) in the logarithmic growth phase were harvested and uniformly mixed with a specified amount of sterile 3@10 bioink, resulting in a cell density of ≈2.5 × 10^6^ cells mL^−1^ in the bioink. This cell‐laden bioink was then printed onto glass slides in pre‐designed patterns using the programmable handheld bioprinter in pneumatic driven mode at room temperature. The printed strips were cross‐linked by exposure to 25 mW cm^−^^2^ of 405 nm wavelength blue light for 15 s. After printing, the strips were washed with fresh PBS and incubated in a 37 °C cell culture incubator. Cell viability within the strips was assessed using a live/dead cell staining kit (Beyotime, China). The stained cells were observed under a fluorescence microscopy imaging system (Keyence BZ‐X810, Japan), and three areas were randomly selected for imaging and viability analysis.

### Rat Full‐Thickness Skin Wound Repair In Vivo

All animal studies were conducted in accordance with Animal Ethics Committee of the Shenzhen Institutes of Advanced Technology, Chinese Academy of Sciences (ACUC) regulations. (Ethical approval: SIAT‐IACUC‐230824‐YYS‐ZXL‐A2311). The PRP was obtained based on modifications to reported methods.^[^ ^27^ ^]^ Ten male Sprague‐Dawley (SD) rats, aged 6–8 weeks and weighing ≈300 g each, were selected. The rats were anesthetized with isoflurane, and ≈10 mL of blood was collected per rat using cardiac puncture method. The rat blood was received in PRP‐specific collection tubes (Sanli, China) containing separating gel, gently inverted 3–5 times to mix with sodium citrate anticoagulant. After allowing it to stand undisturbed at room temperature for 15–20 min, the tubes were centrifuged at a speed of 3200 r min^−1^ for 12 min. The upper two‐thirds of the plasma volume was discarded, while the remaining plasma was thoroughly mixed with the white membrane layer on top of the separating gel. This mixture yielded the PRP. To activate and solidify the PRP, the PRP was mixed with a 10 wt.% calcium chloride solution containing thrombin (250 U mL^−1^ concentration, Beyotime, China) at one‐tenth of its volume. The rats were euthanized by excess anesthesia following the completion of the blood collection. For rat skin wound modeling and repair, eighteen male SD rats, aged 6–8 weeks and weighing ≈300 g each, were randomly divided into three groups with six rats in each group. The rats were anesthetized with isoflurane, and the hair on their backs was removed and disinfected with 75% alcohol. A square full‐thickness skin wound measuring 2.5 cm in length was created in the middle of the back using a surgical scalpel. Five tissue samples were obtained using a 3 mm diameter punch after soaking the excised skin tissue in antibiotic solution. The tissue samples were thoroughly minced to obtain microskin graft, which was then mixed with 0.5 mL of PRP. In the first group, designated as the blank control group, the wound surface was only treated with PBS and bandaged. In the second group, the microskin graft/PRP mixture was dripped onto the wound with a medical syringe, followed by a thrombin solution to promote the gelation of the mixture, and then covered with a bandage. In the third group, the programmable handheld printer equipped with a “thin‐layer” microchannel nozzle was used for application. The lower layer consisted of microskin graft/PRP mixture, while the upper layer contained thrombin solution. After the PRP formed a hydrogel film layer, the wound was bandaged.

### Histological Evaluation

Rats from each group were euthanized via overdose anesthesia. Skin samples from the wound area on the rat's back were excised and fixed in 4% paraformaldehyde solution for 24 h. The wound tissues were then dehydrated through a graded ethanol series, cleared with xylene, and embedded in paraffin. Tissue sections were subsequently prepared and stained with hematoxylin and eosin (H&E). The stained sections were examined and imaged using a light microscope.

### Statistical Analysis

The data were presented as mean ± standard deviation. Statistical significance of differences was determined using one‐way analysis of variance (ANOVA). When *p* < 0.05, it indicates a significant difference between the data. *p* < 0.05 (*), *p* < 0.01 (**), and *p* < 0.001 (***).

## Conflict of Interest

The authors declare no conflict of interest.

## Supporting information



Supporting Information

Supplemental Movie 1

Supplemental Movie 2

## Data Availability

The data that support the findings of this study are available from the corresponding author upon reasonable request.
