# Supplementary material for: A Programmable Handheld Extrusion‐Based Bioprinting Platform for In Situ Skin Wounds Dressing: Balance Mobility and Customizability
Source: Adv Sci (Weinh). 2024 Oct 22;11(46):2405823. doi: 10.1002/advs.202405823 (PMC11633465; doi:10.1002/advs.202405823)
Supplement: Supplementary file 1 — Supporting Information [file ADVS-11-2405823-s002.docx]

Supporting Information

A Programmable Handheld Extrusion-Based Bioprinting Platform for In Situ Skin Wounds Dressing: Balance Mobility and Customizability

Chenmin Wang^#a,b^, Chengwei Hu^#a,c^, Haojin Cheng^#a^, Weichen Qi^#b^, Liangliang Wang^a^，Tianchi Wu^b^, Jun Wu^d^, Xu Cui^a,c^, Jiake Xu^e,f^, Haobo Pan^a,c^, Shaoquan Bian^a,c^, Weijia William Lu^a,b,e^*, Xiaoli Zhao^a,c^*

1. Research Center for Human Tissue and Organs Degeneration, Institute of Biomedicine and Biotechnology, Shenzhen Institute of Advanced Technology, Chinese Academy of Sciences, Shenzhen 518055, China.
2. Department of Orthopaedics and Traumatology, The University of Hong Kong, Hong Kong 999077, China.
3. University of Chinese Academy of Sciences, Beijing 100049, China.
4. Shenzhen Key Laboratory for Innovative Technology in Orthopaedic Trauma, Department of Orthopaedics and Traumatology, The University of Hong Kong-Shenzhen Hospital, Shenzhen 518055, China.
5. Faculty of Pharmaceutical Sciences, Shenzhen University of Advanced Technology, Shenzhen 518055, China.
6. The University of Western Australia, Perth, Western Australia 6000, Australia.

^#^ These authors contributed equally to this work.

*Correspondence: wwlu@siat.ac.cn, zhao.xl@siat.ac.cn


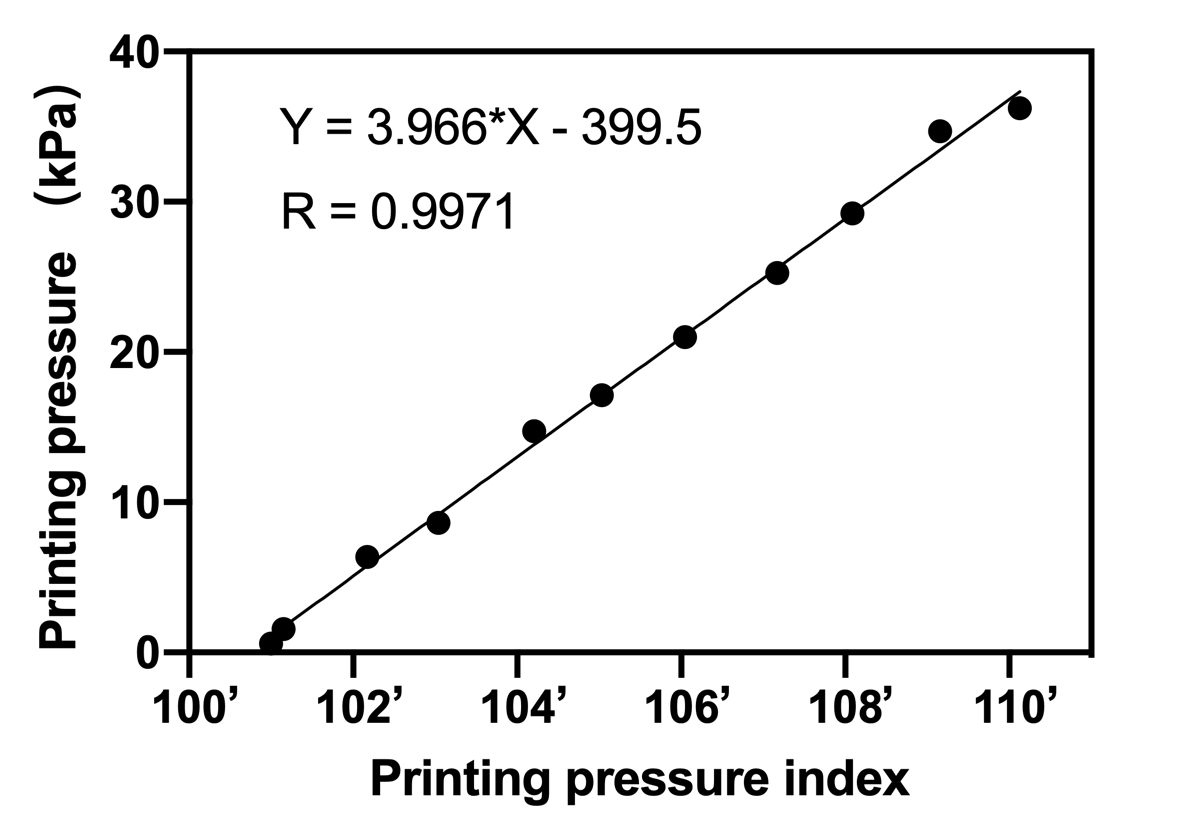


**Figure S1:** Gas pressure calibration. A correlation chart between the printing pressure index in the smartphone Web App and the actual extrusion pressure achieved under the bioink pneumatic extrusion mode.


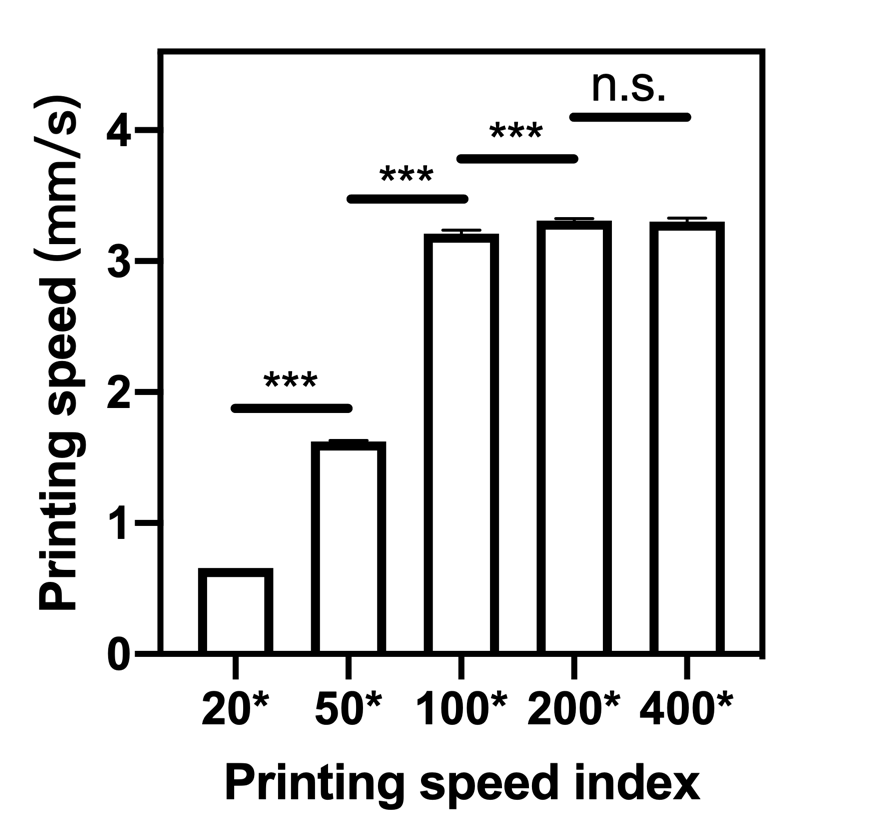


**Figure S2:** Printing speed calibration. A correlation chart depicting the relationship between printing speed index entered into the smartphone Web App and the actual printing speed achieved.


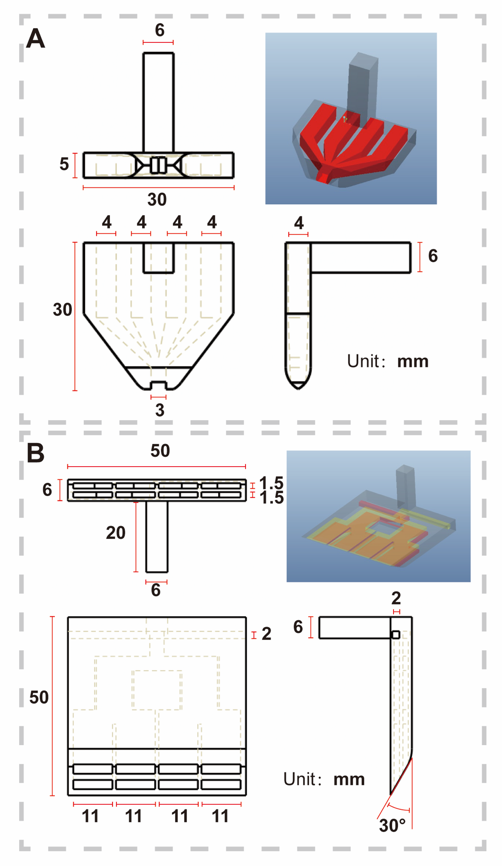


**Figure S3:** Design schematics of microchannel nozzles. Orthographic projections and three-dimensional renderings of the "all-in-one"(A) and "thin-layer"(B) microchannel nozzles.

**
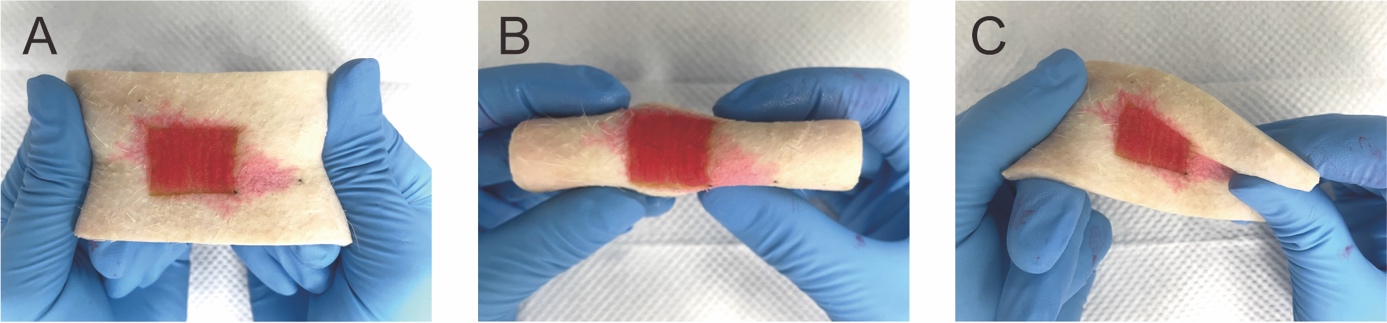
**

**Figure S4** Adhesion of in-situ printed bioink on porcine skin wounds. The hydrogel layer formed by the bioink remained firmly attached to the porcine skin under conditions of stretching (A), bending (B), or twisting (C), without detachment.

**
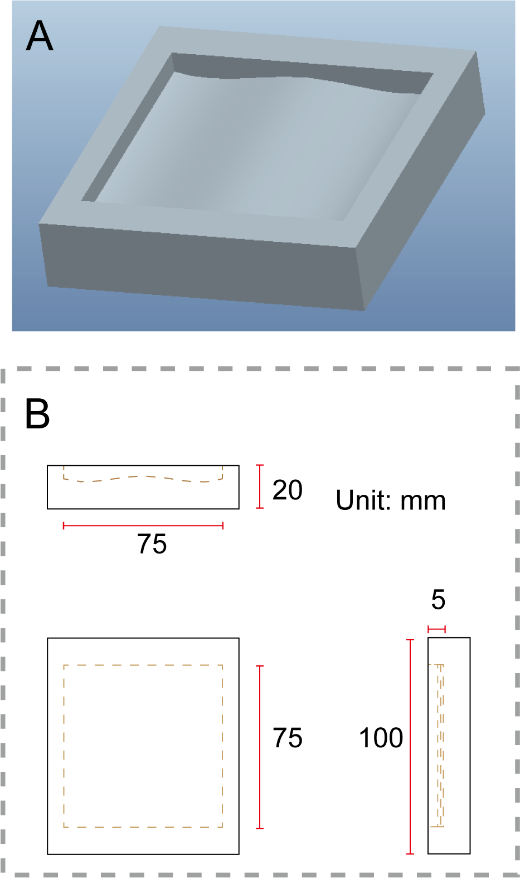
**

**Figure S5** Demonstration of multi-layer bioink printing on a simulated human full-thickness skin wound model. (A) A 3D conceptual design of the simulated human full-thickness skin wound model, depicting the thickness and irregular surface characteristics. (B) An annotated three-view diagram of the model.

**
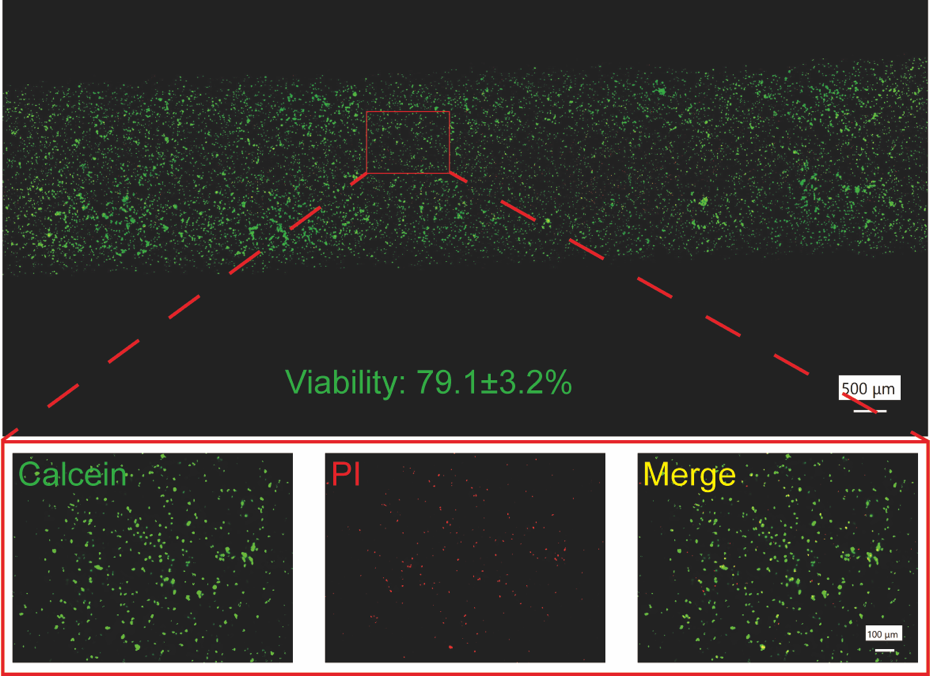
**

**Figure S6** Cell printing using the programmable handheld bioprinter. One day after printing, cells within the strips were processed for live/dead staining, with live cells labelled green with calcein and dead cells labelled red with PI. Cell survival was maintained at 79.1 ± 3.2% in three randomly selected regions.

**
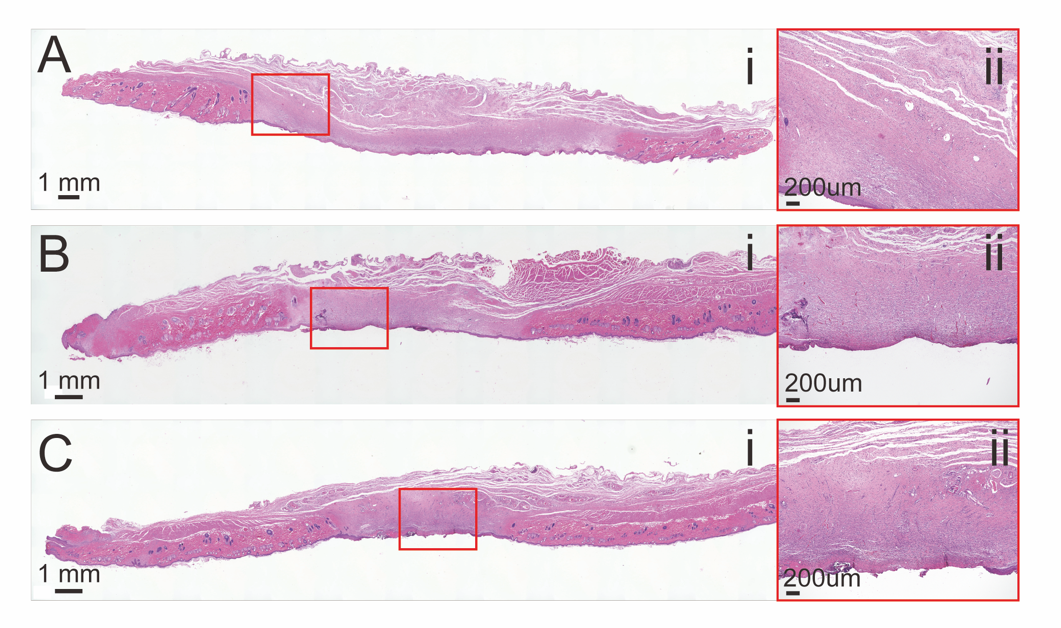
**

**Figure S7** Histological evaluation of rat skin wounds. H&E stained images of Blank group (A), dropping group (B), and printing group (C) on the 15^th^ day after surgery, where (ii) of each group was the locally enlarged image in the red box in the corresponding panorama (i).

**Movie S1:** Demonstration of the programmable handheld printer operating under three distinct printing path modes: "line printing", "S-Curve printing", and "Self-Designed".

**Movie S2:** Printing demonstrations of the programmable handheld printer equipped with "all-in-one" and "thin-layer" microchannel nozzles, respectively.
